# Tracking Changes in Primary Care Clinicians’ Medicaid Participation Using Novel Methods

**DOI:** 10.3390/ijerph22091339

**Published:** 2025-08-27

**Authors:** Mandar Bodas, Qian Luo, Anushree Vichare

**Affiliations:** Fitzhugh Mullan Institute for Health Workforce Equity, Department of Health Policy and Management, Milken Institute of Public Health, George Washington University, Washington, DC 20052, USA

**Keywords:** health workforce, Medicaid, underserved communities, access to care, health policy

## Abstract

Medicaid enrollees often face barriers to care due to inconsistent clinician participation. This study investigates how primary care clinicians’ engagement with Medicaid evolved from 2016 to 2019, focusing on those already serving Medicaid patients at baseline. Using longitudinal data from the Transformed Medicaid Statistical Information System Analytic Files (TAFs), we analyzed changes in the number of unique Medicaid enrollees served annually by 220,665 clinicians across 40 states. We defined major increases or decreases as changes exceeding 90% of baseline enrollee volume and examined associations with clinician, practice, and state-level characteristics. The results show that while about 60% of clinicians maintained stable enrollee volumes, nearly 20% experienced substantial increases or decreases. Higher baseline enrollee volume and affiliation with community health centers were associated with greater stability, while rural practice settings were linked to higher odds of major decreases. These findings underscore the dynamic nature of Medicaid participation and highlight the need for policies that support consistent clinician engagement. As Medicaid programs face potential funding cuts and eligibility changes, tracking participation trends and reinforcing stable provider networks will be critical to safeguarding access to care.

## 1. Introduction

Medicaid is the largest public health insurance program in the United States, jointly funded by the federal government and individual state governments [1]. More than 70 million individuals are enrolled in Medicaid, including low-income adults, children, pregnant women, older adults, and people with disabilities [2]. Although Medicaid is intended to ensure access to essential health services for vulnerable populations, many Medicaid enrollees continue to face persistent barriers to accessing timely care [3]. Chief among these barriers is a shortage of clinicians who consistently serve Medicaid patients [4,5]. Nationally, about 20% of primary care clinicians do not see any Medicaid patients, and only 40% serve more than 100 Medicaid enrollees each year [6]. These gaps in clinician participation in Medicaid can lead to long wait times, delayed treatment, and poor health outcomes [7]. Economic factors are central to this issue: Medicaid reimbursement rates are often substantially lower than those offered by commercial insurers, making it financially challenging for clinicians to sustain Medicaid practice, particularly when caring for patients with complex medical and social needs [5]. Administrative burdens, such as prior authorization requirements and billing complexities, further discourage participation [8]. From the clinician perspective, these challenges can render Medicaid practice unsustainable, especially in smaller or independent settings. Moreover, inconsistent participation undermines continuity of care, which is essential for managing chronic conditions and preventing avoidable emergency department visits [9,10]. Addressing these barriers through targeted policy interventions is critical to ensuring that Medicaid enrollees receive timely, high-quality primary care.

A stable primary care workforce that meaningfully participates in Medicaid is critical to ensuring access to care. This is especially important considering federal access standards, such as a recent CMS rule that requires a maximum 15-day wait time for primary and obstetrical care [11]. Clinician participation in Medicaid is shaped by several factors, including low reimbursement rates, administrative burden, and the complexity of addressing patients’ health-related social needs [12]. These factors not only affect whether a clinician chooses to serve Medicaid patients at all, but also how many they serve. Importantly, Medicaid participation is not static and the number of Medicaid enrollees served by a clinician can change significantly from year to year. Both individual and systemic factors impact clinicians’ level of Medicaid participation over time.

While prior research has provided valuable cross-sectional snapshots of Medicaid participation, often using clinician surveys, these studies are limited in scope [6,13,14]. Most studies examine whether a clinician accepted any Medicaid patients at a single point in time, rather than tracking how participation evolved among those who already accepted Medicaid patients. As a result, we know relatively little about how the number of Medicaid patients served by an individual clinician changes over time, or how such patterns vary by clinician specialty, practice setting, or policy environment. Understanding these dynamics is essential for developing policies that support a robust and responsive Medicaid primary care workforce.

To address this gap, we conducted a longitudinal analysis of primary care clinician participation with Medicaid using multi-state data from the Transformed Medicaid Statistical Information System (T-MSIS) Analytic Files (TAF) [15]. We focused on clinicians who were already serving Medicaid enrollees in 2016. Using that year as a baseline, we tracked changes in the number of unique Medicaid enrollees they served annually from 2016 to 2019.

The primary objective was to identify the proportion of clinicians who already participated in Medicaid and those who experienced substantial increases or decreases in the number of enrollees served compared to the baseline. The secondary objective was to examine how these proportions varied by clinician specialty and profession. We also sought to uncover key clinician-, practice-, or state-level characteristics associated with substantial increases or decreases in the number of enrollees served.

Our focus on clinicians already participating in Medicaid is unique and allows for the estimation of how stable their engagement with Medicaid remained over time. Study findings can offer policymakers a clearer picture of how clinician participation in Medicaid ‘fluctuates’ and exactly who is most likely to maintain or shift their level of participation. This information can be combined with workforce entry and exit projections to inform efforts aimed at strengthening Medicaid access and ensuring continuity of care for enrollees.

## 2. Materials and Methods

### 2.1. Data

This study relied on 2 main datasets: (1) 2016 to 2019 Transformed Medicaid Statistical Information System Technical Analytic Files (T-MSIS TAF) and (2) 2016 to 2019 National Plan and Provider Enumeration System (NPPES) [16]. These datasets were combined at the provider level using the National Provider Identifier (NPI). Specifically, we used multiple TAF files including the Other Services (OT) and Annual Provider files, as well as the Demographic and Eligibility (DE) file. NPPES was used as the primary source for clinician location and specialty classification due to its completeness. Individual NPIs were distinguished from institutional NPIs using NPPES taxonomy and entity type codes. In addition, data from KFF was used for state Medicaid policy characteristics.

### 2.2. Sample

TAF data provided comprehensive information on Medicaid claims, allowing for a detailed analysis of clinicians’ Medicaid participation. Primary care clinicians in the sample included Nurse Practitioners (NPs), Physician Associates (PAs) and physicians who specialized in Family Medicine (FP), General Internal Medicine (IM), Obstetrics/Gynecology (OBGYN), and pediatrics.

We excluded all clinicians who did not participate in Medicaid in 2016 from analyses. We also excluded clinicians who changed their state of location during the study period, since moving to a different state could impact the number of Medicaid enrollees served by a clinician.

Differences between specialties and professions exist in terms of the first year when a clinician is enrolled in the NPPES and the first year when they start billing for patients’ office visits. For example, medical school graduates might receive an NPI either at the end of their medical training or their first year of residency. In contrast, nursing students receive an NPI after they graduate. To account for such variations, we excluded clinicians who were enumerated in the NPPES after 2012. Thus, all clinicians in the sample had at least 3 years to ‘settle down’ their practice. We also excluded clinicians who served more than 2500 Medicaid enrollees in a single year during the study period. Serving such a large number of Medicaid enrollees is unusual and it is likely that such clinicians have peculiar practice patterns that are not representative of the primary care workforce [17]. Finally, clinicians practicing in states with poor-quality TAF data were also excluded. As described in prior workforce studies that used TAF, data quality was assessed by analyzing the number of claims from a state that had missing clinician identifiers [18]. After applying all exclusions, the final sample size included 220,665 primary care clinicians, from forty states (excluded states and territories—Arkansas, California, Delaware, District of Columbia, Florida, Indiana, Maine, Minnesota, New Hampshire, Pennsylvania, Rhode Island, and Tennessee). The sample selection process is described in Figure S1 of the Supplementary Materials.

### 2.3. Outcomes

Using TAF claims data, we operationalized Medicaid participation as the annual count of Medicaid enrollees served by each clinician. This approach differs from certain prior studies that relied on the survey question, ‘Do you accept Medicaid patients?’ and coded the response as a binary outcome [19]. More specifically, we examined the change in the number of unique Medicaid enrollees served office visits by a clinician in a year, relative to the 2016 baseline. We focused on office visits because they represent the principal setting in which clinicians provide care to Medicaid enrollees, as opposed to episodic or acute encounters occurring in other settings such as emergency department or inpatient admissions. Thus, our measure of clinician participation in Medicaid is both clinically relevant and consistent with the delivery of primary care. Claims for office visits were identified in TAF using a combination of Current Procedural Terminology (CPT) and Healthcare Common Procedure Coding System (HCPCS) codes for evaluation and management (E&M) services (see Table S1 for a list of codes in the Supplementary Materials).

Notably, the analysis did not focus on changes in the count of visits but instead on the unique number of enrollees served office visits (enrollee count) each year. Figure S2 in the Supplementary Materials includes an illustration based on data from a random sample of 500 clinicians. It shows that the number of enrollees served can vary significantly year-on-year. To allow for a meaningful analysis, we categorized continuous changes in the number of enrollees served into groups such as large increases or decreases. This is a well-established practice for simplifying interpretation and for highlighting important patterns in the data [20].

For each year after the baseline, we calculated the absolute change in the number of enrollees served relative to the previous year. For example, in 2017, the change was calculated as the difference between the number of enrollees served in 2017 and 2016, while in 2018, it was the difference between 2018 and 2017 and so on. The total net change across all years was then computed as the sum of these year-over-year differences. Aggregating changes over multiple time periods and expressing them as a proportion of a baseline value is a straightforward way to assess the impact of associated factors [21]. Accordingly, we expressed the total net change as a proportion of the baseline number of enrollees in 2016.

We defined two binary outcomes. The first outcome, ‘experiencing a major increase from the baseline’, was coded as 1 if the total net change was positive and greater than 90% of the baseline. In other words, these clinicians experienced a more than 90% increase from the 2016 baseline in the number of enrollees they served over the next three years. It was coded 0 otherwise (for more clarification, see two outcome coding examples in the Supplementary Materials).

Similarly, the second binary outcome, ‘experiencing a major decrease from the baseline’, was coded as 1 if clinicians experienced a more than 90% decrease from the 2016 baseline in the number of enrollees served. In this case, the total net change was negative. All other clinicians (those who did not meet either of these criteria) were classified as experiencing no major change from the baseline. The 90% threshold was selected to capture substantial changes while maintaining interpretability and sample size balance, ensuring that only clinicians with truly noteworthy shifts in enrollee numbers were flagged. Analytical findings remained robust when alternate thresholds of 50 and 75% were used in sensitivity analysis (see Table S2, Figures S3 and S4 of the Supplementary Materials).

Together, these analytical steps allowed us to comprehensively assess overall patterns of change in the number of enrollees served by clinicians over the study period.

### 2.4. Controls

In all statistical models, we controlled for a comprehensive set of clinician-, practice-, and state-level factors. The main clinician-level factor included the number of enrollees served at the baseline (in 2016). We grouped clinicians into the following categories: those serving 1 to 10 enrollees, those serving 11 to 50 enrollees, those serving 51 to 100 enrollees, and those serving more than 100 enrollees. Similar categories have been used in prior research to discern clinician engagement with Medicaid programs. We also accounted for clinician sex.

At the practice level, we controlled for whether a clinician’s practice was located in a rural area and whether it was a Community Health Center (CHC). The county in which each clinician practiced was categorized as rural based on Rural-Urban Continuum Codes (RUCC) [22]. Clinicians practicing in counties with RUCC codes of 4 or higher were classified as rural. We determined whether an individual NPI was associated with a CHC using a proprietary dataset which combined information from the Health Resources and Services Administration’s (HRSA) Uniform Data System (UDS) and the Centers for Medicare & Medicaid Services’ (CMS) Provider Enrollment, Chain, and Ownership System (PECOS) [23].

State-level controls included indicators of Medicaid expansion before the baseline year (2016), expansion during the study period (2016 to 2019) or no expansion at any time [24]. To account for differences in the scope of practice regulations for nurse practitioners across states, we coded each state as having full practice authority, reduced practice authority, or restricted practice authority, and included this variable in our models. These regulations are likely to impact how NPs are allowed to practice in a state and may also have spillover impacts on other specialties and professions [25]. We included a control for the proportion of a state’s Medicaid population covered under fee-for-service arrangements, which served as an indirect measure of the penetration of managed care contracting in state Medicaid programs. Most states deliver Medicaid benefits primarily through managed care organizations (MCOs), which receive a set payment per enrollee and are responsible for contracting with clinicians and managing care [26]. By controlling for the proportion of enrollees remaining in fee-for-service, we account for variation in how much of a state’s Medicaid population is subject to managed care’s influence on clinician networks. MCOs determine which clinicians are included in their networks, which in turn can affect the number of enrollees that each clinician serves.

### 2.5. Statistical Analysis

We began by describing the characteristics of the clinicians in our sample and summarizing state-level trends in the proportion of clinicians who experienced a major increase or decrease in the number of enrollees served.

Multivariable logistic regression models were used to understand characteristics associated with study outcomes. For each specialty and profession, we ran two models—the first compared clinicians experiencing a major increase to those that had stable enrollee volumes, and the second compared clinicians experiencing a major decrease to those that had stable enrollee volumes. While this method does not fully replicate a multinomial logistic regression model with three outcome categories (major increase, major decrease, and stable enrollee volume as the reference), it provides a practical and interpretable alternative for assessing associations relative to the stable enrollee volume reference group. Stratifying models by specialty and profession captured substantial heterogeneity in clinician characteristics and practice contexts that might otherwise require including interaction terms in the analysis. Thus, it simplified coefficient interpretation while reducing the need for complex interaction structures. All models adjusted for clustering of errors at the state level using the state identifier from the baseline year.

To evaluate model specification and to address multicollinearity, we conducted supplemental analyses with elastic net regularized logistic regression. This approach allowed us to examine which predictors remained in the models after applying elastic net regularization, which combines both lasso and ridge penalties. A 2-sided significance threshold of *p* = 0.025 was used. Stata MP, version 18 (StataCorp LLC., College Station, TX, USA) was used to conduct all analyses [27].

## 3. Results

### 3.1. Descriptive Findings

In our analytical sample of 220,556 primary care clinicians, the proportion of clinicians experiencing a major increase in the number of Medicaid enrollees served between 2016 and 2019 ranged from 14% (Peds) to 24% (PAs) (Table 1). The highest proportion of clinicians experiencing a major decrease were NPs (26%) and PAs (24%). Approximately two-thirds of clinicians reported no major change in Medicaid enrollee volume, with the highest proportion observed among Peds (70%) and OBGYNs (68%). Baseline enrollee volume varied by specialty: 72% of Peds and 54% of FPs served more than 100 enrollees, while a relatively smaller share of NPs (36%) and PAs (35%) did so. Table S4 of the Supplementary Materials describes state-level variation in the proportion of clinicians in each category.

### 3.2. Regression Results

Multivariate logistic regression models showed that clinicians serving more Medicaid enrollees at baseline were significantly less likely to experience a major increase in enrollee volume (Table 2). Compared with those serving 1 to 10 enrollees, clinicians serving more than 100 had lower odds ratios (ORs) of 0.05 (95% Confidence Interval [CI], 0.04–0.07) for NPs, 0.05 (95% CI, 0.04–0.07) for FPs, 0.07 (95% CI, 0.04–0.11) for IMs, 0.04 (95% CI, 0.03–0.06) for OBGYNs, 0.05 (95% CI, 0.04–0.06) for PAs, and 0.06 (95% CI, 0.03–0.09) for Peds.

Female clinicians had higher odds of experiencing a major increase in enrollee volume among several specialties, including OBGYNs (OR, 1.15; 95% CI, 1.02–1.31), Peds (OR, 1.18; 95% CI, 1.03–1.36), and FPs (OR, 1.11; 95% CI, 1.00–1.23). However, female NPs (OR, 0.78; 95% CI, 0.70–0.87) and PAs (OR, 0.86; 95% CI, 0.79–0.93) had lower odds of experiencing a major increase. Practicing in a rural area was associated with higher odds of major increases for NPs (OR, 1.51; 95% CI, 1.22–1.87) and PAs (OR, 1.20; 95% CI, 1.03–1.40), but not for other groups. Clinicians practicing at community health centers had consistently higher odds of experiencing major increases across all specialties and professions, including IMs (OR, 2.75; 95% CI, 1.80–4.19), Peds (OR, 2.30; 95% CI, 1.62–3.27), and FPs (OR, 2.18; 95% CI, 1.55–3.08).

Odds of experiencing a major decrease in enrollee volume were also significantly lower for clinicians with higher baseline enrollee volume (Table 3). Compared with those serving 1 to 10 enrollees, clinicians serving more than 100 had ORs of 0.18 (95% CI, 0.15–0.21) for NPs, 0.13 (95% CI, 0.10–0.15) for FPs, 0.10 (95% CI, 0.08–0.12) for IMs, 0.11 (95% CI, 0.09–0.13) for OBGYNs, 0.19 (95% CI, 0.16–0.22) for PAs, and 0.08 (95% CI, 0.07–0.09) for Peds.

Female OBGYNs had lower odds of experiencing a major decrease (OR, 0.69; 95% CI, 0.63–0.76), while female Peds (OR, 0.93; 95% CI, 0.89–0.98) and PAs (OR, 0.88; 95% CI, 0.82–0.95) also had slightly lower odds. Practicing in a rural area was associated with higher odds of major decreases for most groups, including IMs (OR, 1.56; 95% CI, 1.40–1.73), Peds (OR, 1.26; 95% CI, 1.09–1.46), and FPs (OR, 1.22; 95% CI, 1.11–1.35). Clinicians affiliated with community health centers had lower odds of experiencing major decreases across all specialties, including NPs (OR, 0.19; 95% CI, 0.13–0.27), IMs (OR, 0.51; 95% CI, 0.34–0.78), and Peds (OR, 0.35; 95% CI, 0.20–0.63).

### 3.3. Sensitivity Analysis

Table S3 of the Supplementary Materials include descriptive results from alternate specifications of study outcomes. As expected, the proportion of clinicians classified as experiencing a major increase or decrease in the number of enrollees served rose as the threshold became less stringent. For example, at the 90% threshold, 17.6% clinicians in the full sample experienced a major increase and 20.3% a major decrease. These proportions rose to 19.1% and 23.7% at the 75% threshold, and to 22.6% and 30.6% at the 50% threshold, respectively. Figures S3 and S4 of the Supplementary Materials include a plot of regression coefficients for select predictors. Three panels in each figure include results from the same logistic regression model, but with the outcome defined using three thresholds: 90% (main analysis), 75%, and 50%. The direction and relative magnitude of the coefficients remain consistent across all thresholds, indicating that the regression findings are robust to alternate specifications of study outcomes.

Results from supplemental analyses are included in Tables S4 and S5 of the Supplementary Materials. We used elastic net regularization methods to help select the predictors that are most important (model specification) and to prevent the model from being overly influenced by highly correlated variables (multicollinearity). The elastic net regularization method does this by combining two techniques: lasso and ridge regression. Lasso helps select a smaller set of important predictors by setting some coefficients to zero (in other words, removing some variables from the model). On the other hand, ridge helps reduce the effect of highly correlated predictors by shrinking their coefficients toward zero, but rarely setting them to zero. We used the elasticnet command in Stata, which chose the predictors most important for explaining study outcomes. Categories of baseline number of enrollees served were retained as predictors in nearly all models, except for the model predicting experiencing a major increase for pediatricians. However, a few other predictors such as practice rurality were dropped for certain specialties and professions. This indicates that the baseline number of enrollees served is generally a robust and important predictor across most specialties and professions.

## 4. Discussion

This study offers a novel longitudinal perspective on Medicaid participation among primary care clinicians, focusing on those who were already engaged with the program in the baseline year and how their participation changed over the course of the next three years. Findings showed that while around 60% maintained stable enrollee volumes, a significant proportion experienced major changes. Such fluctuation in clinician engagement with Medicaid is a critical metric for understanding the reliability of access to care for Medicaid enrollees. Our results underscore the need to explore new methods of clinician participation in Medicaid [28]. Even after using a generous threshold of 90%, nearly one in five clinicians experienced a major reduction in the number of Medicaid enrollees served, while a similar proportion saw major increases. These shifts suggest that access to care may be more volatile than previously understood. Regression results highlight the importance of baseline enrollee volume as a predictor of stability, suggesting that clinicians with higher initial engagement with Medicaid are more likely to sustain participation.

Our method, which classified major changes in Medicaid participation based on cumulative net change over multiple years, provides a more nuanced perspective than simply comparing participation in the baseline year to that in the final year. This approach captures cumulative shifts in clinician engagement and avoids the statistical inefficiencies associated with single-year comparisons.

Differences in participation trends by specialty and profession were particularly noteworthy. Pediatricians and OBGYNs demonstrated more stable enrollee volumes compared to NPs and PAs. Several factors may explain these patterns. For instance, the nature of care provided by pediatricians and OBGYNs, such as routine well-child visits and prenatal care, is often unavoidable [18]. This may contribute to more consistent Medicaid engagement. In contrast, the practice of ‘incident to’ or indirect billing may obscure the true volume of Medicaid enrollees served by NPs and PAs, leading to apparent fluctuations in their participation [29]. In such cases, services delivered by non-physician clinicians (such as NPs and PAs) may be billed under a supervising physician’s NPI, rather than the individual who actually delivered the care. This billing practice can distort clinician-level measures of Medicaid participation and may disproportionately affect observed trends for NPs and PAs.

Practice setting also played a key role. Clinicians affiliated with CHCs were more likely to experience increases and less likely to experience decreases in enrollee volume. These findings highlight the importance of CHCs in caring for Medicaid populations and institutional support in sustaining individual clinicians’ Medicaid participation. Conversely, practicing in rural areas was associated with greater odds of major decreases, pointing to geographic differences that merit further investigation.

Medicaid programs could benefit from tracking clinician participation over time and identifying those with consistent engagement. To that end, our findings on state-level trends in the proportion of clinicians with stable enrollee volumes can be used. Such data could inform managed care contracting strategies, including requirements to include ‘stable participant’ clinicians in plan networks. Additionally, state workforce retention efforts could be prioritized in rural areas and among clinician types with higher volatility.

Study findings are also relevant to proposed federal Medicaid policy changes that could significantly reduce Medicaid enrollment and funding [30,31,32]. Such changes are likely to exacerbate the patterns observed in this study. Clinicians who already serve smaller Medicaid panels may see further reductions in enrollee volume, increasing the risk of disengagement. Administrative burdens associated with eligibility redeterminations and coverage lapses could further destabilize state Medicaid populations and thereby clinician participation. This is particularly worrying for NPs and PAs, who already face structural challenges in maintaining consistent Medicaid engagement. Moreover, reductions in federal funding may constrain the ability of CHCs and other safety-net providers to retain clinicians or expand services, undermining one of the few settings associated with more stable Medicaid participation. If enacted, these policy changes could lead to a contraction of the Medicaid primary care workforce, making it even more critical to identify and support clinicians who demonstrate consistent engagement.

### Limitations

This study has several limitations that should be considered when interpreting the findings. First, the use of ‘incident to’ or indirect billing may lead to underestimation of the number of clinicians providing care [29]. Second, the study period (2016 to 2019) predates the COVID-19 pandemic. While this allows for analysis during a relatively stable environment, it does not capture the post-pandemic policy landscape. Third, although the study includes a large sample across 40 states, it excludes states with poor-quality TAF data. As a result, findings may not be generalizable to all states or to the national Medicaid clinician workforce. Fourth, the analysis focuses exclusively on clinicians who were already participating in Medicaid in 2016. It does not account for new entrants or exits from the Medicaid program during the study period. Future research should explore these dynamics to provide a more comprehensive understanding of workforce turnover and access implications. Finally, while the models adjust for a wide range of clinician-, practice-, and state-level factors, they do not include granular controls for local-level demand for services or clinician supply. These unmeasured factors may influence changes in enrollee volume and should be explored in future work using geospatial or market-level analyses.

## 5. Conclusions

Our analysis reveals that Medicaid participation among primary care clinicians is dynamic, with substantial year-to-year variation even among those who initially served Medicaid enrollees. These fluctuations have important implications for access to care and continuity. As policymakers consider significant changes to Medicaid financing, it is essential to monitor clinician engagement and design policies that support a stable and resilient primary care workforce.

The relevance of this study’s findings is heightened in light of proposed Medicaid budget cuts, which include stricter eligibility requirements and reduced federal funding. These changes could further destabilize clinician participation, particularly among those already serving smaller Medicaid panels or practicing in rural areas. Tailored strategies, especially those that reinforce the role of CHCs, address geographic disparities, and reduce administrative burdens, will be vital to ensuring that Medicaid enrollees continue to have access to timely and consistent care.

## Figures and Tables

**Table 1:** Characteristics of Primary Care Clinicians in the Sample.

	Nurse Practitioners	Family Medicine Physicians	Internal Medicine Physicians	OBGYNs	Pediatricians	Physician Associates
Clinicians (N)	43,936	52,390	52,755	20,289	28,212	22,974
Major increase from the baseline	9828 (22%)	7312 (14%)	8869 (17%)	3345 (16%)	3841 (14%)	5566 (24%)
Major decrease from the baseline	11,247 (26%)	10,231 (20%)	10,122 (19%)	3159 (16%)	4500 (16%)	5497 (24%)
No major change	22,861 (52%)	34,847 (67%)	33,764 (64%)	13,785 (68%)	19,871 (70%)	11,911 (52%)
Baseline number of enrollees served						
1 to 10	10,508 (24%)	7051 (13%)	12,765 (24%)	2815 (14%)	2764 (10%)	6346 (28%)
11 to 50	11,093 (25%)	9347 (18%)	14,303 (27%)	4169 (21%)	2745 (10%)	5625 (24%)
51 to 100	6310 (14%)	7580 (14%)	9092 (17%)	3413 (17%)	2289 (8%)	3033 (13%)
100 or more	16,025 (36%)	28,412 (54%)	16,595 (31%)	9892 (49%)	20,414 (72%)	7970 (35%)
Clinician Characteristics						
Female	40,942 (93%)	20,899 (40%)	19,633 (37%)	11,630 (57%)	17,710 (63%)	14,920 (65%)
Practice Characteristics						
Practiced in rural areas	9059 (21%)	13,106 (25%)	5243 (10%)	2353 (12%)	2630 (9%)	4679 (20%)
Practice at a Community Health Center	1939 (4%)	2469 (5%)	939 (2%)	662 (3%)	1028 (4%)	664 (3%)
State Policy Characteristics						
Full nurse scope of practice	11,461 (26%)	13,860 (26%)	11,178 (21%)	4608 (23%)	5953 (21%)	7668 (33%)
Reduced nurse scope of practice	19,515 (44%)	21,558 (41%)	24,172 (46%)	8800 (43%)	12,227 (43%)	7893 (34%)
Restricted nurse scope of practice	12,960 (29%)	16,972 (32%)	17,405 (33%)	6881 (34%)	10,032 (36%)	7413 (32%)
No Medicaid expansion	15,735 (36%)	19,784 (38%)	14,479 (27%)	7105 (35%)	9569 (34%)	9250 (40%)
Medicaid Expansion before 2016	26,955 (61%)	30,722 (59%)	36,783 (70%)	12,512 (62%)	17,568 (62%)	13,459 (59%)
Medicaid Expansion during 2016 to 2019	1246 (3%)	1884 (4%)	1493 (3%)	672 (3%)	1075 (4%)	265 (1%)

**Table 2:** Odds of Experiencing a Major Increase in the Number of Medicaid Enrollees Served, 2016–2019.

	Nurse Practitioners	Family Medicine Physicians	Internal Medicine Physicians	OBGYNs	Physician Associates	Pediatricians
N	32,689	42,159	42,633	17,130	17,477	23,712
Clinician Characteristics						
Baseline enrollees 1 to 10 (reference)						
Baseline enrollees 11 to 50	0.33 **	0.35 **	0.37 **	0.39 **	0.33 **	0.55 **
	(0.30–0.38)	(0.28–0.42)	(0.31–0.44)	(0.33–0.47)	(0.29–0.38)	(0.40–0.76)
Baseline enrollees 51 to 100	0.17 **	0.18 **	0.17 **	0.16 **	0.18 **	0.32 **
	(0.14–0.21)	(0.13–0.24)	(0.12–0.24)	(0.13–0.21)	(0.14–0.22)	(0.23–0.46)
Baseline enrollees more than 100	0.05 **	0.05 **	0.07 **	0.04 **	0.05 **	0.06 **
	(0.04–0.07)	(0.04–0.07)	(0.04–0.11)	(0.03–0.06)	(0.04–0.06)	(0.03–0.09)
Clinician Characteristics						
Male (reference)						
Female	0.78 **	1.11 *	1.10	1.15 *	0.86 **	1.18 *
	(0.70–0.87)	(1.00–1.23)	(1.00–1.21)	(1.02–1.31)	(0.79–0.93)	(1.03–1.36)
Practice Characteristics						
Practice in a rural area	1.51 **	1.18	1.08	1.19	1.20 *	1.39
	(1.22–1.87)	(0.95–1.48)	(0.78–1.50)	(0.84–1.68)	(1.03–1.40)	(0.95–2.03)
Practice at a Community Health Center	1.53 **	2.18 **	2.75 **	1.97 **	2.16 **	2.30 **
	(1.13–2.07)	(1.55–3.08)	(1.80–4.19)	(1.30–2.99)	(1.37–3.39)	(1.62–3.27)
State Policy Characteristics						
Full nurse scope of practice (reference)						
Reduced nurse scope of practice	1.37	1.21	1.41 *	1.72 **	1.19	1.48
	(0.97–1.94)	(0.80–1.83)	(1.03–1.92)	(1.14–2.58)	(0.96–1.49)	(0.88–2.50)
Restricted nurse scope of practice	1.48	1.30	2.28	2.67 *	1.40	2.18
	(0.94–2.32)	(0.53–3.21)	(0.87–6.01)	(1.01–7.06)	(0.84–2.33)	(0.64–7.35)
No Medicaid expansion (reference)						
Medicaid Expansion before 2016	1.09	1.22	1.64	1.46	1.35	1.13
	(0.66–1.81)	(0.60–2.51)	(0.73–3.65)	(0.67–3.20)	(0.94–1.94)	(0.38–3.41)
Medicaid Expansion during 2016 to 2019	0.45 **	2.78 **	3.07 **	0.95	0.97	0.42 **
	(0.34–0.59)	(1.57–4.95)	(1.61–5.84)	(0.63–1.44)	(0.75–1.25)	(0.27–0.66)
Percentage of Medicaid population in Fee For Service	2.39	3.00	2.11	2.62	1.22	1.65
	(0.67–8.51)	(0.77–11.76)	(0.68–6.52)	(0.75–9.17)	(0.77–1.93)	(0.31–8.87)

Confidence intervals in parentheses, ** *p* < 0.01, * *p* < 0.05.

**Table 3:** Odds of Experiencing a Major Decrease in the Number of Medicaid Enrollees Served, 2016–2019.

	Nurse Practitioners	Family Medicine Physicians	Internal Medicine Physicians	OBGYNs	Physician Associates	Pediatricians
N	34,108	45,078	43,886	16,944	17,408	24,371
Clinician Characteristics						
Baseline enrollees 1 to 10 (reference)						
Baseline enrollees 11 to 50	0.33 **	0.35 **	0.37 **	0.39 **	0.33 **	0.55 **
	(0.30–0.38)	(0.28–0.42)	(0.31–0.44)	(0.33–0.47)	(0.29–0.38)	(0.40–0.76)
Baseline enrollees 51 to 100	0.17 **	0.18 **	0.17 **	0.16 **	0.18 **	0.32 **
	(0.14–0.21)	(0.13–0.24)	(0.12–0.24)	(0.13–0.21)	(0.14–0.22)	(0.23–0.46)
Baseline enrollees more than 100	0.05 **	0.05 **	0.07 **	0.04 **	0.05 **	0.06 **
	(0.04–0.07)	(0.04–0.07)	(0.04–0.11)	(0.03–0.06)	(0.04–0.06)	(0.03–0.09)
Clinician Characteristics						
Male (reference)						
Female	0.78 **	1.11 *	1.10	1.15 *	0.86 **	1.18 *
	(0.70–0.87)	(1.00–1.23)	(1.00–1.21)	(1.02–1.31)	(0.79–0.93)	(1.03–1.36)
Practice Characteristics						
Practice in a rural area	1.51 **	1.18	1.08	1.19	1.20 *	1.39
	(1.22–1.87)	(0.95–1.48)	(0.78–1.50)	(0.84–1.68)	(1.03–1.40)	(0.95–2.03)
Practice at a Community Health Center	1.53 **	2.18 **	2.75 **	1.97 **	2.16 **	2.30 **
	(1.13–2.07)	(1.55–3.08)	(1.80–4.19)	(1.30–2.99)	(1.37–3.39)	(1.62–3.27)
State Policy Characteristics						
Full nurse scope of practice (reference)						
Reduced nurse scope of practice	1.37	1.21	1.41 *	1.72 **	1.19	1.48
	(0.97–1.94)	(0.80–1.83)	(1.03–1.92)	(1.14–2.58)	(0.96–1.49)	(0.88–2.50)
Restricted nurse scope of practice	1.48	1.30	2.28	2.67 *	1.40	2.18
	(0.94–2.32)	(0.53–3.21)	(0.87–6.01)	(1.01–7.06)	(0.84–2.33)	(0.64–7.35)
No Medicaid expansion (reference)						
Medicaid Expansion before 2016	1.09	1.22	1.64	1.46	1.35	1.13
	(0.66–1.81)	(0.60–2.51)	(0.73–3.65)	(0.67–3.20)	(0.94–1.94)	(0.38–3.41)
Medicaid Expansion during 2016 to 2019	0.45 **	2.78 **	3.07 **	0.95	0.97	0.42 **
	(0.34–0.59)	(1.57–4.95)	(1.61–5.84)	(0.63–1.44)	(0.75–1.25)	(0.27–0.66)
Percentage of Medicaid population in Fee for Service	2.39	3.00	2.11	2.62	1.22	1.65
	(0.67–8.51)	(0.77–11.76)	(0.68–6.52)	(0.75–9.17)	(0.77–1.93)	(0.31–8.87)

Confidence intervals in parentheses, ** *p* < 0.01, * *p* < 0.05.

## Data Availability

The data used in this study was obtained by the authors from the Centers for Medicare and Medicaid Services (CMS), the data use agreement signed by the authors disallows them from sharing it.

## Supplementary Materials

The following supporting information can be downloaded at: https://www.mdpi.com/article/10.3390/ijerph22091339/s1, Figure S1: Sample creation; Table S1: List of codes used to identify office visits; Figure S2: Illustration of fluctuation in the number enrollees served each year by individual clinicians; Table S2: Descriptive results from alternate specifications of study outcomes at 50% and 75% thresholds; Figure S3: comparing regression results for alternate specifications of major increase; Figure S4: comparing regression results for alternate specifications of major decrease; Table S3: State-level trends; Table S4: Results from elastic net regularization logistic regressions for major increase; Table S5: Results from elastic net regularization logistic regressions for major decrease.

## Abbreviations

The following abbreviations are used in this manuscript:

TAF	Transformed Medicaid Statistical Information System Analytic Files (TAFs)
NPPES	National Plan and Provider Enumeration System
NPI	National Provider Identifier
NP	Nurse Practitioners
FP	Family Physicians
IM	Internal Medicine
OBGYN	Obstetrics/Gynecology
PA	Physician Associates
