# Supplementary material for: Tracking Changes in Primary Care Clinicians’ Medicaid Participation Using Novel Methods
Source: Int J Environ Res Public Health. 2025 Aug 27;22(9):1339. doi: 10.3390/ijerph22091339 (PMC12470067; doi:10.3390/ijerph22091339)
Supplement: Supplementary file 1 [file ijerph-22-01339-s001.zip › ijerph-3742098-supplementary.pdf]

Supplement

Figure S1 Sample creation

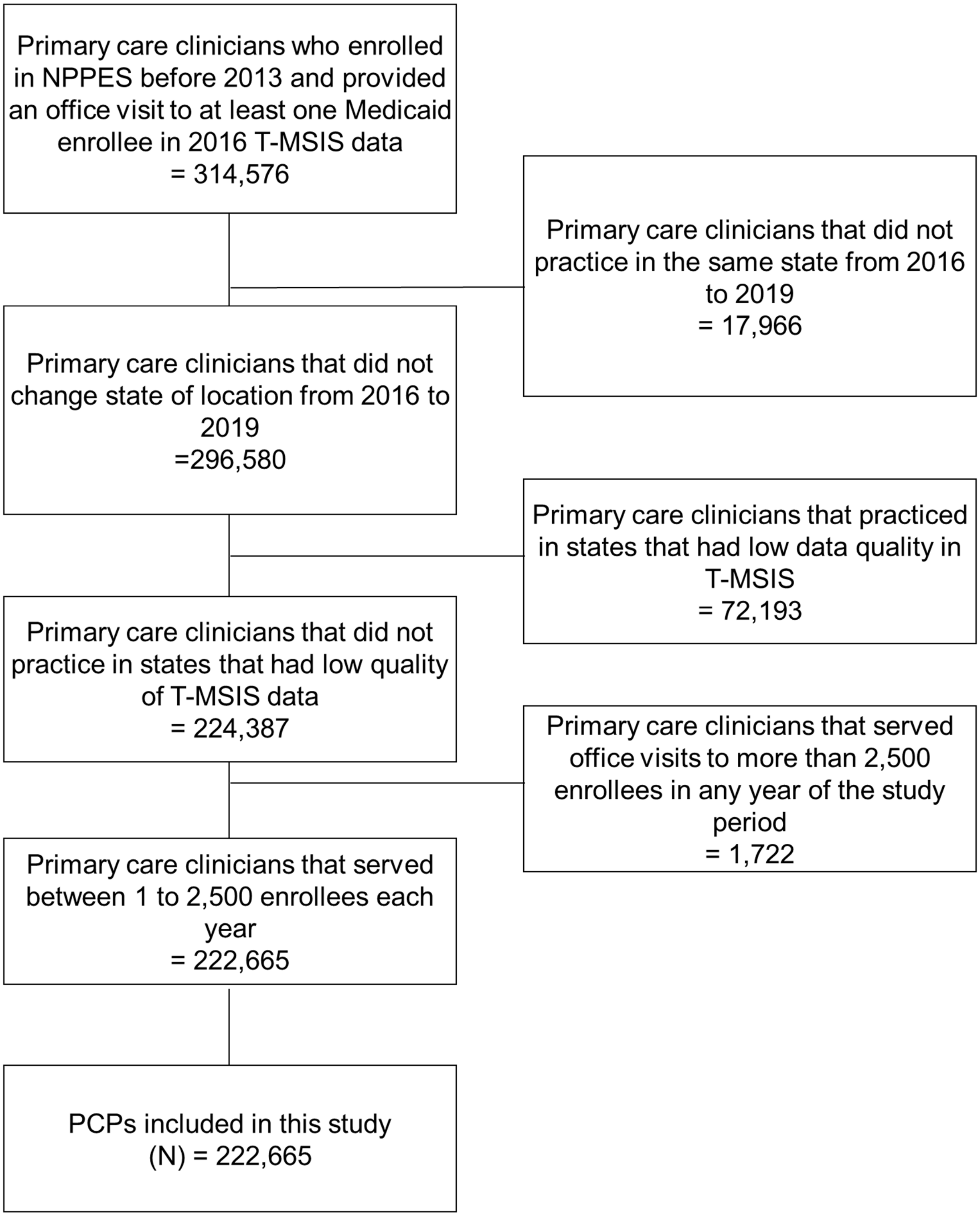

Table S1 List of codes used to identify office visits

0500F	99215
99201	99354
99202	99355
99203	99366
99204	99367
99205	99391
99381	99392
99382	99393
99383	99394
99384	99395
99385	99396
99386	99397
99387	99401
G0101	99402
G0245	99403
G0248	99404
G0402	99411
G0466	99412
G0469	99429
0502F	G0246
0503F	G0247
1000F	G0250
2000F	G0420
95115	G0421
95117	G0463
99058	G0467
99211	G0468
99212	G0470
99213	G0473
99214	G2083

Figure S2 Illustration of fluctuation in the number enrollees served each year by individual clinicians

The goal of this study was to leverage multiple years of Medicaid claims data (2016-2019 TAF) to understand how clinicians alter their Medicaid participation. To examine whether a clinicians' participation increased or decreased in each year, we first quantified the unique number of Medicaid enrollees that they served with an office visit. The following illustration depicts results from plotting this measure for a random sample of 500 clinicians.

Each line represents an individual clinician.

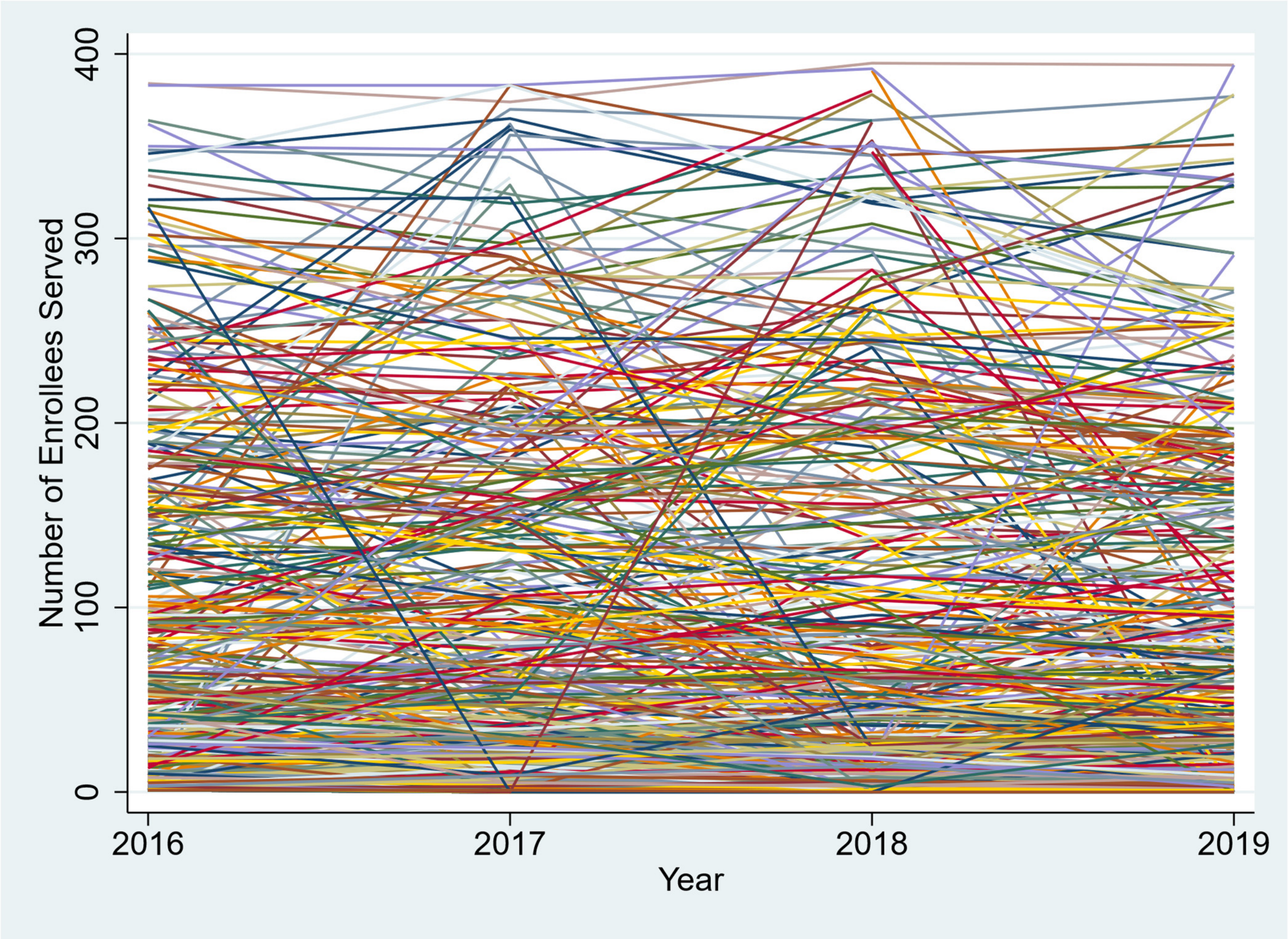

As seen in the image, there are endless patterns which cannot be easily discerned into simple categories of – experienced increase or decrease in the number of enrollees served compared to the baseline.

We explored several different methods to study clinicians' participation in Medicaid to ensure that our analytic choices are an accurate representation of what we observed in the data.

See coding examples in the next section to understand how we created study outcomes -

Coding example

We illustrate the outcome coding process below with two examples –
Example 1: Substantial Enrollee Growth (Clinician A)
Enrollee Counts:

Year	Enrollees
2016	100
2017	130
2018	160
2019	200

Calculation:

1. Year-over-year changes:
   - δ_2017 = 130 – 100 = 30
   - δ_2018 = 160 – 130 = 30
   - δ_2019 = 200 – 160 = 40
2. Total net change (Δ):
   - Δ = 30 + 30 + 40 = 100
3. Proportion of baseline:
   - Δ / baseline = 100 / 100 = 1.0 (100%)
4. Outcome:
   - Since 100% > 90%, clinician A experienced major increase (1)

Example 2: Moderate Enrollee Growth (Clinician B)
Enrollee Counts:

Year	Enrollees
2016	80
2017	90
2018	95
2019	100

Calculation:

1. Year-over-year changes:
   - δ_2017 = 90 – 80 = 10
   - δ_2018 = 95 – 90 = 5
   - δ_2019 = 100 – 95 = 5
2. Total net change (Δ):
   - Δ = 10 + 5 + 5 = 20
3. Proportion of baseline:
   - Δ / baseline = 20 / 80 = 0.25 (25%)
4. Outcome:
   - Since 25% < 90%, clinician B was coded as not having experienced a major increase (0)

Table S2 Descriptive results from alternate specifications of study outcomes at 50% and 75% thresholds

Outcome	Full Sample	NP	FP	IM	OBGYN	PA	Peds
Major Increase (90%)	17.6%	22.4%	14.0%	16.8%	16.5%	24.2%	13.6%
Major Increase (75%)	19.1%	23.9%	15.3%	18.5%	18.1%	25.7%	15.2%
Major Increase (50%)	22.6%	27.4%	18.1%	22.4%	22.1%	28.9%	18.8%
Major Decrease (90%)	20.3%	25.6%	19.5%	19.2%	15.6%	23.9%	16.0%
Major Decrease (75%)	23.7%	29.4%	23.5%	22.0%	18.5%	28.0%	18.4%
Major Decrease (50%)	30.6%	36.2%	32.0%	28.4%	25.9%	35.0%	23.2%

Figures S3 comparing regression results for alternate specifications of major increase

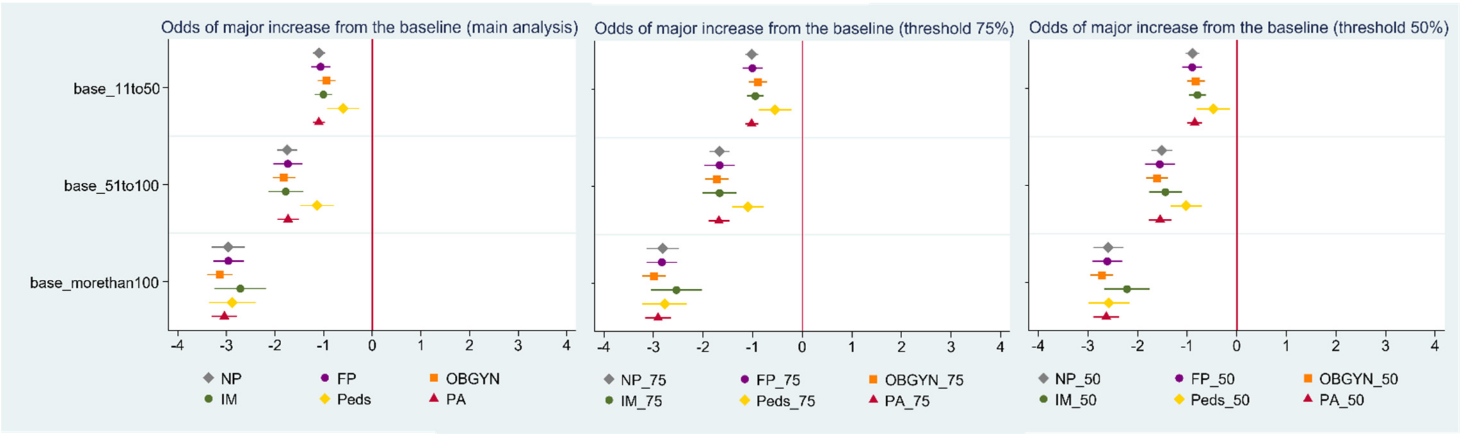

Figures S4 comparing regression results for alternate specifications of major decrease

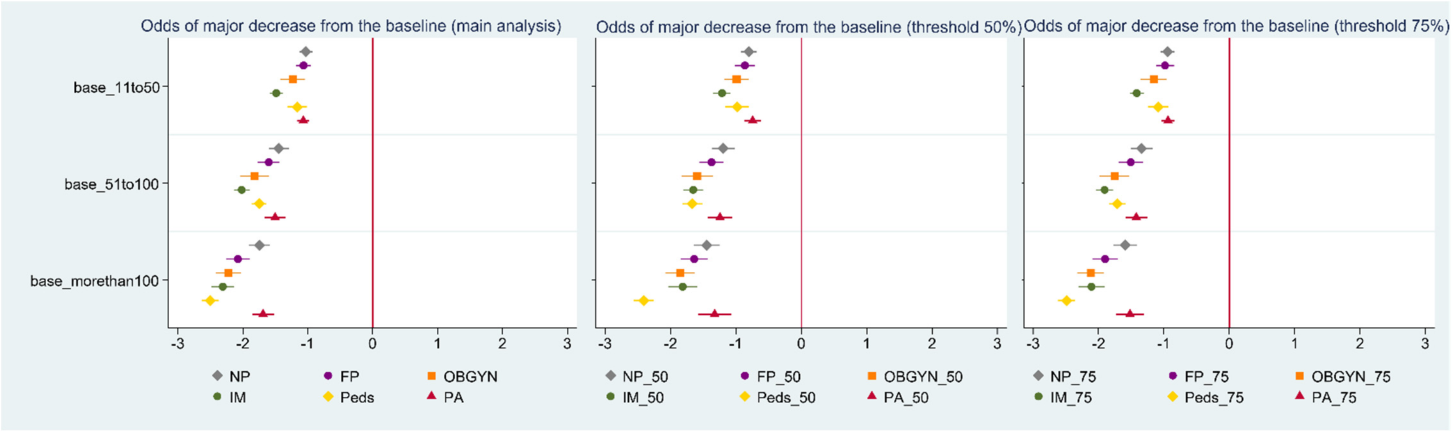

Table S3 State-level trends

State	Major increase from the baseline	Major decrease from the baseline	No major change	Total
Alabama	371 (11%)	734 (21%)	2,404 (69%)	3,509 (100%)
Alaska	115 (10%)	247 (22%)	750 (67%)	1,112 (100%)
Arizona	830 (15%)	1,545 (28%)	3,198 (57%)	5,573 (100%)
Colorado	1,185 (19%)	1,314 (21%)	3,686 (60%)	6,185 (100%)
Connecticut	663 (14%)	937 (20%)	3,118 (66%)	4,718 (100%)
Georgia	1,125 (14%)	1,640 (21%)	5,034 (65%)	7,799 (100%)
Hawaii	113 (9%)	206 (17%)	900 (74%)	1,219 (100%)
Idaho	211 (12%)	272 (15%)	1,350 (74%)	1,833 (100%)
Illinois	1,660 (14%)	2,744 (23%)	7,311 (62%)	11,715 (100%)
Iowa	691 (19%)	626 (17%)	2,411 (65%)	3,728 (100%)
Kansas	466 (15%)	735 (24%)	1,815 (60%)	3,016 (100%)
Kentucky	694 (14%)	865 (17%)	3,539 (69%)	5,098 (100%)
Louisiana	973 (22%)	713 (16%)	2,674 (61%)	4,360 (100%)
Maryland	864 (15%)	1,179 (21%)	3,599 (64%)	5,642 (100%)
Massachusetts	4,155 (42%)	1,536 (15%)	4,237 (43%)	9,928 (100%)
Michigan	1,761 (14%)	2,555 (21%)	7,974 (65%)	12,290 (100%)
Mississippi	1,779 (65%)	456 (17%)	489 (18%)	2,724 (100%)
Missouri	1,999 (36%)	923 (17%)	2,608 (47%)	5,530 (100%)
Montana	172 (14%)	274 (22%)	789 (64%)	1,235 (100%)
Nebraska	325 (13%)	382 (16%)	1,719 (71%)	2,426 (100%)
Nevada	290 (15%)	498 (25%)	1,200 (60%)	1,988 (100%)
New Jersey	1,454 (21%)	1,303 (19%)	4,099 (60%)	6,856 (100%)
New Mexico	417 (18%)	518 (22%)	1,423 (60%)	2,358 (100%)
New York	3,557 (16%)	4,498 (20%)	14,233 (64%)	22,288 (100%)
North Carolina	1,556 (14%)	2,162 (19%)	7,574 (67%)	11,292 (100%)
North Dakota	213 (21%)	175 (18%)	611 (61%)	999 (100%)
Ohio	1,514 (11%)	2,293 (17%)	9,420 (71%)	13,227 (100%)
Oklahoma	565 (15%)	729 (20%)	2,372 (65%)	3,666 (100%)
Oregon	656 (12%)	1,077 (20%)	3,737 (68%)	5,470 (100%)
South Carolina	673 (16%)	785 (19%)	2,770 (66%)	4,228 (100%)
South Dakota	250 (22%)	204 (18%)	669 (60%)	1,123 (100%)
Texas	2,541 (14%)	4,705 (26%)	10,848 (60%)	18,094 (100%)
Utah	1,121 (45%)	454 (18%)	915 (37%)	2,490 (100%)
Vermont	200 (20%)	186 (19%)	612 (61%)	998 (100%)
Virginia	1,593 (24%)	1,491 (22%)	3,551 (54%)	6,635 (100%)
Washington	902 (10%)	1,833 (20%)	6,299 (70%)	9,034 (100%)
West Virginia	280 (14%)	514 (26%)	1,184 (60%)	1,978 (100%)
Wisconsin	788 (10%)	1,361 (17%)	5,833 (73%)	7,982 (100%)
Wyoming	39 (19%)	87 (41%)	84 (40%)	210 (100%)

Tables S4 Results from elastic net regularization logistic regressions for major increase

	(1)<br>NP	(2)<br>FP	(3)<br>IM	(4)<br>OBGYN	(5)<br>Peds
Baseline enrollees 11 to 50	0.338***	0.340***	0.373***	0.392***	
	(0.0205)	(0.0354)	(0.0360)	(0.0373)	
Baseline enrollees 11 to 50	0.175***	0.177***	0.176***	0.163***	0.461***
	(0.0176)	(0.0291)	(0.0360)	(0.0196)	(0.0691)
Baseline enrollees more than 100	0.0523***	0.0526***	0.0717***	0.0433***	0.0799***
	(0.00901)	(0.00956)	(0.0214)	(0.00576)	(0.0189)
Female	0.773***				
	(0.0457)				
Practice in a rural area	1.511***				
	(0.165)				
Practice at a Community Health Center	1.525**	2.215***	2.794***	1.970**	2.323***
	(0.236)	(0.385)	(0.577)	(0.419)	(0.482)
Percentage of Medicaid population in Fee For Service	1.389	1.128	1.218	1.692*	0.942
	(0.249)	(0.244)	(0.162)	(0.352)	(0.317)
Reduced nurse scope of practice	1.422	1.285	1.810	2.634*	
	(0.334)	(0.422)	(0.708)	(1.293)	
Medicaid Expansion before 2016	1.130			1.458	
	(0.296)			(0.604)	
Restricted nurse scope of practice	2.535	2.681		2.651	
	(1.650)	(1.876)		(1.735)	
Observations	32689	42159	42633	17130	23712

Exponentiated coefficients; Standard errors in parentheses

* $p < 0.05$, ** $p < 0.01$, *** $p < 0.001$

Note: No lambda was selected for models for PAs, and were excluded

Tables S5 Results from elastic net regularization logistic regressions for major decrease

	(1)<br>NP	(2)<br>FP	(3)<br>IM	(4)<br>PA	(5)<br>Peds
Baseline enrollees 11 to 50	0.358***	0.345***	0.226***	0.344***	0.313***
	(0.0182)	(0.0207)	(0.0116)	(0.0164)	(0.0239)
Baseline enrollees 11 to 50	0.236***	0.203***	0.133***	0.223***	0.174***
	(0.0181)	(0.0175)	(0.00844)	(0.0183)	(0.0102)
Baseline enrollees more than 100	0.175***	0.126***	0.0993***	0.185***	0.0818***
	(0.0138)	(0.0120)	(0.00866)	(0.0159)	(0.00542)
Female	0.898*	0.970		0.881**	
	(0.0391)	(0.0324)		(0.0345)	
Practice in a rural area	1.131*	1.220***	1.554***	1.057	1.264**
	(0.0707)	(0.0591)	(0.0814)	(0.0745)	(0.0934)
Practice at a Community Health Center	0.191***	0.379***	0.515**	0.307***	0.352***
	(0.0345)	(0.0819)	(0.109)	(0.0656)	(0.104)
Percentage of Medicaid population in Fee For Service	1.179	0.954	1.348*	1.190	1.018
	(0.167)	(0.133)	(0.164)	(0.194)	(0.126)
Reduced nurse scope of practice		0.862*	0.882	1.147	1.013
		(0.0559)	(0.0703)	(0.159)	(0.0611)
Medicaid Expansion before 2016		1.147*	1.063	1.206	
		(0.0669)	(0.0884)	(0.176)	
Restricted nurse scope of practice			0.916	1.210	
			(0.0840)	(0.207)	
Observations	34108	45078	43886	17408	24371

Exponentiated coefficients; Standard errors in parentheses

* $p < 0.05$, ** $p < 0.01$, *** $p < 0.001$

Note: No lambda was selected for models for OBGYNs, and were excluded
